# Dysregulation of immune monocyte responses during sepsis

**DOI:** 10.1186/cc9679

**Published:** 2011-03-11

**Authors:** D Fiume, G Caiazza, V Tsekeuli, A Sinistro, C Almerighi, F Calo'-Carducci, E Baffari, A Bergamini, S Natoli, F Leonardis

**Affiliations:** 1 Policlinico Roma Tor Vergata, ICU, Rome, Italy

## Introduction

Despite intense efforts, sepsis remains a serious clinical problem, accounting for thousands of deaths every year. Many findings have shown that immune dysfunction in septic patients plays a very important role. Thus, a better understanding of the basic immune alterations in sepsis is needed to appropriately direct therapy. Here we sequentially measured TNFα, IL-1B, IL-6 and IL-10 *de novo *synthesis by monocytes via multiparametric flow cytometry and monocyte expression of surface molecules that allow effective antigen presentation, in patients with severe sepsis and septic shock up to 12 days after admission.

## Methods

Twenty-five patients and 15 healthy, age and sex matched control subjects were enrolled. Each patient met the following criteria: an identifiable site of infection; two or more systemic inflammatory response syndrome criteria. Septic shock was defined as severe hypotension that lasts 1 hour, despite adequate fluid resuscitation and pharmacologic intervention with vasopressor agents. *Cell stimulation *PBMC from patients and controls were cultured for 18 hours in the presence of 100 ng/ml LPS and analysed by FACS to determine cell surface antigen expression and intracellular cytokine production.

## Results

### Cytokine production by monocytes during sepsis

Monocytes from septic patients produced significantly higher amounts of IL-1B, TNFα and IL-6, but not IL-10 as compared with controls. In addition, monocytes from patients with septic shock responded to LPS stimulation with increased IL-1B, TNFα and IL-6 production with respect to cells from patients without septic shock.

### Serum cytokine levels

All cytokines were readily detectable in septic patients.

### Effect of sepsis on surface molecule expression

Monocyte CD80, CD86 and HLA-DR expression was significantly decreased in patients with sepsis as compared with healthy subjects. As opposed, the expression of ILT4 was significantly increased in septic patients as compared with healthy controls.

## Conclusions

It has been postulated that the immune response in sepsis represents the interplay of two contrasting phenomena: the early systemic inflammatory response syndrome followed by the late appearance of a compensatory anti-inflammatory response syndrome. The findings reported here suggest a scenario, characterized by the contemporary development of an intense proinflammatory reaction and a marked alteration of the phenotype of antigen-presenting cells.

